# Color Development in Carotenoid-Enriched Bigels: Effects of Extraction Method, Saponification, and Oleogel-to-Hydrogel Ratios on CIELAB Parameters

**DOI:** 10.3390/gels11100823

**Published:** 2025-10-14

**Authors:** Caroline Ramos-Souza, Daniel Henrique Bandoni, Veridiana Vera de Rosso

**Affiliations:** Nutrition and Food Service Research Center, Federal University of São Paulo (UNIFESP), Santos 11015-020, SP, Brazil; caroline.silva@unifesp.br (C.R.-S.); dbandoni@unifesp.br (D.H.B.)

**Keywords:** bigel, carotenoid, color, CIELAB, dye, hydrogel, oleogel

## Abstract

Bigels are promising delivery systems for bioactive compounds, combining the properties of hydrogels and oleogels. Pequi carotenoids, characterized by their natural yellow fluorescence, hold potential to replace the artificial dye tartrazine in foods while simultaneously enhancing their functional properties. This study developed food-grade bigels with varying oleogel-to-hydrogel ratios (40%, 60%, 80% OG) to assess the pigmentation capacity of pequi carotenoid extracts. Hydrogel contained agar and xanthan gum, while oleogel comprised beeswax, lecithin, sunflower oil, and 400 μg/100 g carotenoid extract. Bigel color was analyzed using the CIELAB system. Linear and multiple regression models were applied to assess the influence of crosslinking time (1 vs. 12 h), extraction solvent (acetone vs. [BMIM][BF4]), saponification, and oleogel ratio on color parameters. The color of the carotenoid-enriched bigels was mainly influenced by the extraction solvent and the oleogel ratio, while saponification and crosslinking time had only minor impacts. Although changes in L*, a*, and b* were observed across samples, ΔE* values generally reflected low perceptibility. Notably, more evident color differences were associated with variations in solvent type and oleogel ratio. These findings contribute to a better understanding of how formulation parameters influence the pigmentation behavior and support the development of natural, visually appealing functional foods.

## 1. Introduction

Color perception, including the ability to distinguish hues and intensities, is a fundamental cognitive skill [1] that strongly shapes food perception and acceptance, influencing expectations of quality, freshness, and flavor [2]. Even subtle color variations can affect consumer preference, highlighting its importance as a key sensory attribute [1]. With the increasing demand for plant-based and naturally colored foods, understanding color behavior in complex food matrices is essential [3], although achieving stable and predictable color outcomes with natural pigments such as carotenoids remains a challenge [4].

Carotenoids, fat-soluble pigments responsible for reddish-yellow hues, owe their color to a conjugated double bond system that forms a π-electron resonance chromophore [5]. This chromophore defines the visible absorption spectrum, and variations in its structure or concentration directly affect the perceived color of food products [6]. Beyond their coloring properties, carotenoids contribute nutritionally through provitamin A activity, supporting cell differentiation, immunity, and vision, while non-provitamin A carotenoids may protect against oxidative stress and age-related macular degeneration [7,8,9]. Epidemiological studies have shown that carotenoid-rich diets have been associated with a lower risk of chronic diseases, stimulating research into their distribution and levels across different food matrices [10]. Among natural carotenoid sources, pequi (*Caryocar brasiliense* Camb.), a native fruit from the Brazilian Cerrado biome, stands out for its carotenoid content and intense yellow-orange coloration. Pequi carotenoids were selected as a model in this study due to their visual similarity to tartrazine, an artificial yellow dye widely used in the food industry. The yellow hue is often associated with energy and joy, and artificial dyes are frequently employed in products targeted at children, especially those featuring cartoon characters [11]. This resemblance to tartrazine is further supported by the hue* values in the CIELAB parameters of the pequi carotenoid extracts, which reflect a specific carotenoid composition dominated by molecules with 10 conjugated double bonds (all-*trans*-antheraxanthin) and 11 conjugated double bonds (all-*trans*-zeaxanthin, all-*trans*-β-cryptoxanthin, and all-*trans*-β-carotene) [12]. In addition, the observed color difference (ΔE*) between tartrazine and the pequi extracts confirmed this visual similarity, with ΔE* = 6.93 for carotenoids extracted using acetone and ΔE* = 11.08 for those obtained with the ionic liquid [BMIM][BF4] [12].

These carotenoids naturally occur in free and esterified forms with fatty acids [13]. Saponification is a common analytical technique used to hydrolyze these esters and remove interfering compounds such as triacyl glycerides and chlorophylls, simplifying carotenoid identification in foods. This is particularly useful as a single xanthophyll can form multiple esters with different fatty acids, leading to structurally similar yet distinct compounds that complicate analysis [14]. While advantageous for analytical clarity, saponification does not represent the natural state of carotenoids as consumed, given that dietary sources typically contain xanthophyll esters. Furthermore, the process can induce degradation and isomerization of carotenoids, potentially altering their bioactivity while increasing production costs for commercial formulations [15].

To further explore strategies for preserving the natural profile of carotenoids and their incorporation in esterified forms, this study investigated the integration of free and esterified carotenoids into bigels. These innovative delivery systems, composed of hydrophilic and lipophilic phases, provide a promising platform for carotenoid incorporation [16]. Notably, carotenoids in their free forms have been successfully incorporated into bigels, including astaxanthin [17], lutein [18], lycopene [19], and β-carotene [20,21]. In addition, bigels provide a valuable platform for investigating the optical characteristics of carotenoids through colorimetric analysis. The CIELAB color space, widely used and recommended for industrial applications, especially in the food sector, offers a perceptually uniform scale across the visible spectrum of the human eye, making it suitable for assessing visual attributes critical to consumer perception [22]. By evaluating key parameters such as hue*, Chroma*, and lightness (L*), it is possible to gain insights into how factors like matrix composition (e.g., oleogel-to-hydrogel ratio and crosslinking time) and carotenoid form (free vs. esterified) influence color expression and, consequently, the visual appeal and stability of carotenoid-enriched bigels. Color evaluation is a crucial analytical tool, as it enables the detection of color variations among different formulations and the monitoring of potential changes or degradation over time, thereby providing valuable indications of chemical alterations and overall product quality [23]. Regarding crosslinking time, bigels require sufficient time after preparation to achieve stable network formation. In a bigel based on agar-xanthan gum hydrogel and beeswax oleogel enriched with pitanga carotenoids, 12 h of crosslinking slightly reduced a* and b* values (CIELAB parameters), indicating stabilization of red-yellow hues [24]. Moreover, the decrease in L* values with increasing crosslinking time reflected a slight darkening of the samples, suggesting reduced brightness compared to freshly prepared bigels [24]. With respect to the oleogel-to-hydrogel ratio, increasing the oleogel fraction enhances firmness, viscoelasticity, and carotenoid stability, whereas higher hydrogel content can favor bioactive compound release during digestion [18,19,21]. Incorporation of free lutein into bigels with 75% hydrogel resulted in a greater color difference (ΔE* = 83.59) compared to a 50% hydrogel formulation (ΔE* = 69.45) [18]. Pigment stability during storage further highlights the protective effect of bigels, wherein after 7 days, the color of astaxanthin in corn oil became lighter, indicating degradation, whereas bigels loaded with astaxanthin showed only slight color changes, with higher beeswax concentrations (8%) reducing degradation [17].

Building on this framework, the present study was designed to elucidate the main factors affecting the color parameters of bigels formulated with an agar-xanthan gum hydrogel and a beeswax-based oleogel enriched with pequi carotenoids. Specifically, it investigates how structural and compositional variables, crosslinking time (1 vs. 12 h), oleogel-to-hydrogel ratios (40%, 60%, and 80% OG), and the incorporation of saponified or non-saponified carotenoids extracted using either a conventional volatile solvent (acetone) or an ionic liquid ([BMIM][BF4]), affect color expression within these biphasic systems. Therefore, testing crosslinking times of 1 h and 12 h and oleogel ratios of 40%, 60%, and 80% allows a systematic evaluation of how these parameters modulate color properties, reflecting practical ranges relevant for food applications. By systematically analyzing these factors, this work seeks to bridge existing gaps in colorimetry regarding how matrix architecture, extraction method, and pigment form modulate CIELAB parameters, providing new insights into the optical behavior of carotenoid-enriched bigels, while also linking these variations to functional aspects such as antioxidant stability and sensory attributes like consumer acceptance.

## 2. Results and Discussion

### 2.1. Pequi Carotenoid Profile

An interpretation of the CIELAB colorimetric analyses is not very easy to give, both because only a few data are available in literature about colorimetric studies on bigel enriched with carotenoids [18] and also because the obtained colorimetric parameters are deeply influenced by several factors, including the carotenoid profile. Thus, the carotenoids’ colors are intimately related to the molecular structures, so correct identification is essential for carotenoid analysis [25]. The UV/Vis spectrum represents the presence of the long chromophore of conjugated double bonds (c.d.b.), with at least 7 c.d.b needed for a carotenoid to have a perceptible color [26].

**Figure 1:**
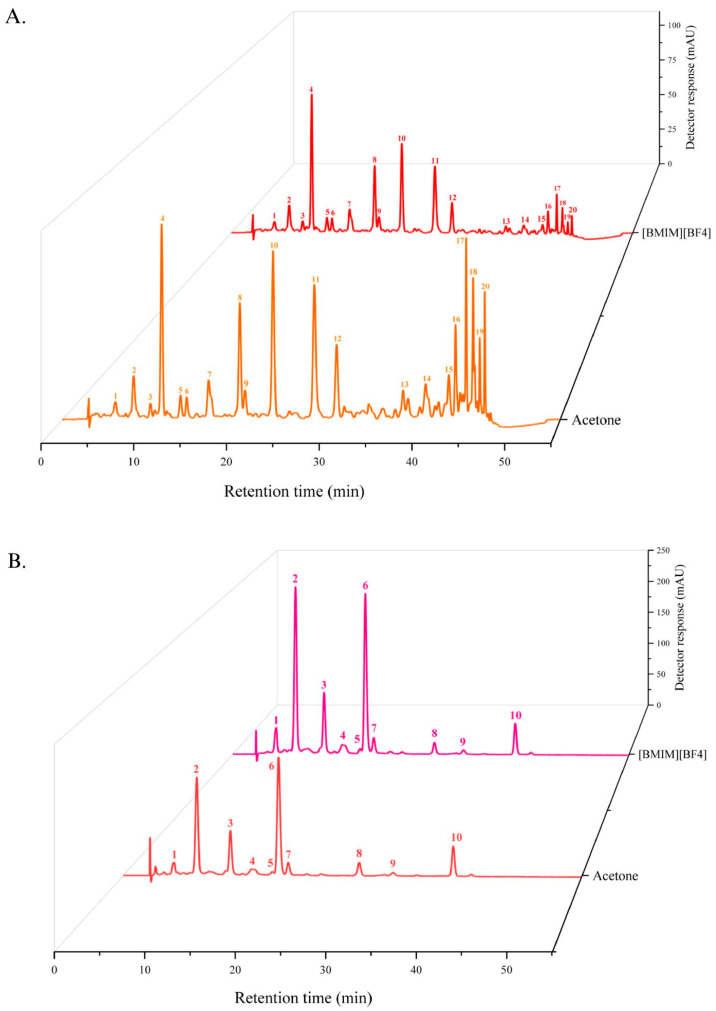
Chromatograms of carotenoid-enriched bigels with (**A**) Non-saponified carotenoids and (**B**) Saponified carotenoids.

The pequi carotenoid extract was tentatively identified in its esterified form using HPLC-PAD-MS/MS, with UV/Vis absorption characteristics summarized in Table 1. To the best of our knowledge, such identification has not been reported in the literature. A total of 20 peaks were separated, comprising four free carotenoids and 16 esters, including four in the *cis* configuration and 12 in the *trans* configuration. The free carotenoids were *cis*-antheraxanthin (peak 1), all-*trans*-antheraxanthin (peak 2), all-*trans*-zeaxanthin (peak 3), and all-*trans*-β-carotene (peak 11) (Figure 1A). Free xanthophylls represented 18.46% of the carotenoid extract obtained with acetone and 31.40% of the extract obtained with [BMIM][BF4], while esterified xanthophylls represented 71.31% and 57.45%, respectively. Additionally, the acetone-extracted sample contained 6.22 µg of β-carotene, whereas the [BMIM][BF4]-extracted sample contained 9.76 µg. To further investigate the composition, the extract was subjected to saponification. Figure 1B shows the resulting chromatogram, and Table 2 summarizes the UV/Vis characteristics of the carotenoids in their free (non-esterified) form. The major peaks correspond to all-*trans*-antheraxanthin (10 c.d.b.), 9-*cis*-antheraxanthin (10 c.d.b.), and all-*trans*-zeaxanthin (11 c.d.b.). The carotenoid profile is a key determinant of the visible color of the extract, with β-carotene contributing an orange-yellow hue and its hydroxylated derivatives, the xanthophylls, conferring a pale-yellow coloration [25,27]. Given this compositional background, the next step was to assess how these pigments influenced the color properties of the carotenoid-enriched bigels.

### 2.2. Color of Carotenoid-Enriched Bigels

The values of all the color parameters evaluated in bigels enriched with pequi carotenoids, namely L*, a*, b*, Chroma*, and hue*, are represented in Table 3. High L* values (71.77 ± 0.64 to 79.04 ± 0.05) were recorded across all extracts, indicating relatively light samples. The lowest L* value was observed for the 40% oleogel (OG) bigel crosslinked for 12 h, enriched with saponified carotenoids extracted using [BMIM][BF4], while the highest was for the 80% OG bigel crosslinked for 1 h, enriched with non-saponified carotenoids extracted using acetone. The b* and Chroma* values were nearly identical among the bigels enriched with different types of carotenoids, likely due to the a* values being close to zero. The hue* values observed in the bigels (ranged from 86.87° ± 0.01 to 90.86° ± 0.00) reflect the influence of the carotenoid concentration and structural interactions within the bigel matrix. Notably, the lowest and highest hue* values were observed in acetone-extracted carotenoid-enriched bigels, saponified and crosslinked for 1 h, with 40% OG and 80% OG, respectively. Interestingly, the bigel enriched with carotenoids extracted using [BMIM][BF4], saponified, and crosslinked for 12 h exhibited a hue* value of exactly 90°, representing a pure yellow hue, devoid of red or green tonal influences. These values are characteristic of carotenoid-containing samples, as they predominantly reflect light within the yellow to orange regions of the visible spectrum, corresponding to hue angles between approximately 70° and 110° in the CIELAB color space color [26].

In this context, the first hypothesis evaluated the effect of crosslinking time (1 vs. 12 h) on the CIELAB color parameters of carotenoid-enriched bigels. Crosslinking is a process in which polymer chains are chemically or physically interconnected, contributing to a three-dimensional network formation. In bigels, crosslinking plays a crucial role in defining the structural integrity, mechanical strength, and overall stability of the system [28]. According to the linear regression analysis, no significant differences were observed in the color parameters between 1 and 12 h of crosslinking (*p* > 0.05) (Table 4). These findings suggest that although crosslinking contributes fundamentally to the structural properties of bigels, its impact on the optical characteristics, as measured by CIELAB parameters, appears to be limited. The slight, non-significant reductions observed in lightness (L* value) and yellowness (b* value) after 12 h of crosslinking may indicate a minimal tendency for color modulation associated with network formation. These results are consistent with previous findings, which reported a tendency toward decreased L*, a* and b* values in agar–xanthan gum-beeswax-based bigels containing carotenoids from Brazilian pitanga fruit after 12 h of crosslinking, suggesting the development of less intense red–yellow tones over time [24]. Overall, the absence of statistically significant differences reinforces that, under the evaluated conditions, the crosslinking process does not substantially affect pigment integrity or color stability, indicating that bigels maintain their optical properties during network formation, an important attribute for applications where color uniformity is required.

The second hypothesis evaluated the effect of the solvent used for carotenoid extraction, acetone versus [BMIM][BF4], on the CIELAB color parameters of bigels. Although both extraction methods yielded the same carotenoids, slight differences in the quantitative profiles were observed [12], which could influence the color characteristics of the resulting bigels. The linear regression analysis revealed statistically significant differences in all color parameters when [BMIM][BF4] was used instead of acetone (Table 4). Specifically, bigels incorporated with carotenoids extracted using [BMIM][BF4] showed lower values for lightness (L*), redness (a*), yellowness (b*), and Chroma*, indicating a darker and less intense coloration, while the hue angle (hue*) was significantly higher, suggesting a perceptible shift in color tone. These differences can be attributed to two complementary factors. First, the selective solubility of specific carotenoids in [BMIM][BF4] during extraction altered the overall quantitative profile of the carotenoid pool, yielding a higher proportion of xanthophylls (59.58% vs. 43.06% in acetone extracts [12]). Second, once incorporated into the bigel matrix, xanthophylls may form supramolecular aggregates or interact with biopolymers, modifying light scattering and optical path within the gel network and resulting in reduced lightness and yellowness [29,30]. Therefore, the darker and less yellow appearance of the [BMIM][BF4]-derived bigels likely reflects both the compositional differences induced by solvent selectivity and structural effects within the bigel, rather than the intrinsic hue of the xanthophylls alone.

Regarding the third hypothesis, which evaluated the effect of saponification on the color parameters of carotenoid-enriched bigels, no statistically significant differences were observed between saponified (S) and non-saponified (NS) extracts (Table 4). These results indicate that, under the tested conditions, the saponification process did not substantially affect the visual color attributes of the bigels. This outcome is consistent with expectations, as the chromophore responsible for carotenoid coloration, defined by the conjugated double bond system, remains intact during de-esterification [29,31]. In pitanga carotenoid-enriched bigels, it was reported that hue* values tend to increase with higher concentrations of non-saponified carotenoids (800 μg), whereas saponified extracts display the opposite trend, with hue* decreasing as carotenoid content rises (400 μg) [24]. Although direct comparisons between saponified and non-saponified carotenoid extracts are limited in the literature, the minimal differences observed in CIELAB parameters in the present study likely reflect the preservation of the chromophore structure during saponification, thereby maintaining the overall color of the bigels.

For hypothesis four, the effect of different oleogel-to-hydrogel (OG: HG) ratios (40%, 60%, and 80% OG) on the color parameters of the bigels was investigated (Table 5). Using 40% OG as the reference level, the results demonstrated that increasing the oleogel ratio significantly influenced color attributes. Both 60% and 80% OG led to a significant increase in lightness (L*), with β values of 2.09 and 2.77, respectively (*p* < 0.001 for both). However, a* values significantly decreased with higher OG ratio, indicating a reduction in red tones. For the b* and Chroma* parameters, only the 60% OG formulation showed a significant reduction, while 80% OG did not differ significantly from 40% OG, suggesting a tipping point effect at 60% OG. Hue* values significantly increased for both 60% and 80% OG, suggesting a shift towards more yellowish hues.

**Figure 2:**
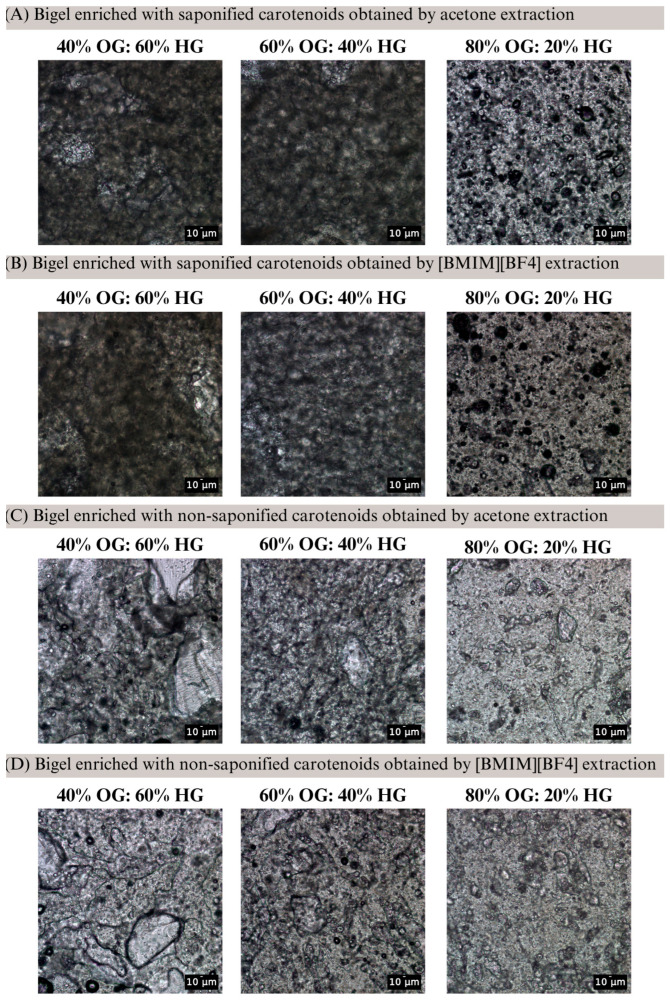
Bright-field micrographs of carotenoid-enriched bigels with different oleogel-to-hydrogel (OG: HG) ratios. (**A**) extracted with acetone and saponified; (**B**) extracted with [BMIM][BF4] and saponified; (**C**) extracted with acetone and non-saponified and (**D**) extracted with [BMIM][BF4] and non-saponified. Images were captured at room temperature (20 ± 2 °C) using bright-field microscopy, illustrating the effect of OG ratio on bigel microstructure and potential implications for carotenoid distribution and color perception. Scale bars = 10 µm.

Bright-field microscopy analysis at 20× magnification revealed that the 40% and 60% OG bigels exhibit a bicontinuous structure, whereas the 80% OG bigel displays a hydrogel-in-oleogel (W/O) structure (Figure 2). In the 40–60% OG bigels, only a few spherical oil droplets were visible, while the water phase did not form discrete droplets but rather merged with the oil phase, resulting in a dense, interconnected three-dimensional network characteristic of a bicontinuous structure. In contrast, the 80% OG micrographs showed a homogeneous, lighter-colored dispersed matrix, with only minimal colorless regions corresponding to the aqueous phase. These observations indicate that the phase behavior of the bigel changes with OG concentration, influencing carotenoid dissolution and dispersion within the matrix, which in turn affects the observed color parameters. Therefore, the pronounced changes in b* and Chroma* at 60% OG can be mechanistically explained by the structural transition to a bicontinuous structure, whereas the 80% OG bigel, with a W/O structure, shows partial recovery of color attributes. These results suggest that the oleogel-to-hydrogel ratio modulates both the microstructure and optical properties of bigels, with 60% OG representing the most impactful ratio for color perception. These findings contrast with previous observations in chlorophyll-enriched bigels, where L*, a*, and b* values remained relatively stable across formulations (20%, 40%, 60%, and 80% OG); yet, over a storage period of seven days, the green coloration was better preserved in formulations with higher hydrogel content, following the order: 20% > 40% > 60% > 80% (OG: HG ratio) [32].

The multivariate analysis revealed that each factor influenced the CIELAB parameters to different extents (Table 6). Among all variables, the [BMIM][BF4] used as a solvent for carotenoid extraction had a consistent and significant effect across all color parameters. Its use resulted in lower L* (β = −2.29), a* (β = −0.70), b* (β = −2.29), and Chroma* (β = −2.31) values, while increasing the hue* (β = +1.35), suggesting a general darkening and desaturation of the yellow color. The oleogel-to-hydrogel ratio also played an important role. The 60% OG condition significantly reduced a* (β = −0.53), b* (−1.97), and Chroma* (−1.99) values compared to 40% OG, while increasing hue*. The 80% OG bigels followed the same trend but did not reach significance for all parameters, suggesting that the most pronounced effect occurs when increasing the oil phase from 40% to 60%. Carotenoid saponification had a significant positive effect only on L* and a negative effect on hue*, while crosslinking after 12 h showed a significant reduction exclusively in the L* parameter (β = −0.66), indicating a small darkening effect potentially associated with increased network density.

To further complement the interpretation of individual color parameters, ΔE* was calculated as a comprehensive metric summarizing the overall color variations across L*, a*, and b* values. While the individual parameters had already indicated differences under various conditions, ΔE* provides an integrated measure of how perceptible these color changes are to the human eye. For the first hypothesis evaluated, related to crosslinking time (1 vs. 12 h), the highest ΔE* observed was for the bigel containing 40% OG enriched with carotenoids extracted using acetone and saponified (ΔE* = 1.90), while the lowest ΔE* was found for the bigel containing 60% OG enriched with carotenoids extracted with acetone and non-saponified (ΔE* = 0.25). Overall, the results indicate that color differences between the 1 and 12 h crosslinked bigels were minimal, with all ΔE* values remaining below 2, within the range generally considered imperceptible or only noticeable upon close observation [33].

When evaluating the impact of the saponification process (saponified vs. non-saponified carotenoids) on the color differences of carotenoid-enriched bigels, it was observed that the changes could be noticeable at a glance for some formulations. Specifically, ΔE* values of 3.74 and 2.14 were found for bigels with 40% and 80% OG, respectively, extracted using acetone, and ΔE* values of 2.75 and 4.30 were found for bigels with 60% and 80% OG extracted with [BMIM][BF4], respectively. In addition, the lowest ΔE* value was recorded for the bigel with 40% OG extracted with [BMIM][BF4], at 0.95.

In contrast, when comparing the color differences between carotenoids extracted with different solvents, the variations were more pronounced. The highest ΔE* value (5.77) was observed for the 80% OG bigel when comparing non-saponified carotenoids extracted with acetone and [BMIM][BF4], while the lowest difference was found for the 60% OG bigel (ΔE* = 2.01). Among saponified bigels, color differences were slightly lower, with ΔE* values ranging from 3.81 (60% OG) to 4.27 (80% OG). When analyzing the extracts without incorporation into bigels, a ΔE* value of 5.81 was reported for pequi carotenoids extracted with acetone and [BMIM][BF4] [12]. This difference is attributed to the distinct quantitative profiles of carotenoids extracted by acetone and [BMIM][BF4], both solvents extract the same carotenoids but in different proportions, which in turn influenced the color characteristics of the resulting extracts and bigels.

For carotenoids extracted with acetone, lower ΔE* values were observed when comparing bigels with 40% and 60% OG (3.39 for saponified and 1.89 for non-saponified carotenoids), compared to the 40% and 80% OG comparison (3.90 and 4.64, respectively). Although the magnitude of the values differs, this trend is consistent with previous observations in bigels enriched with lutein, where variations in the hydrogel-to-oleogel ratio also influenced color perception. Specifically, a higher hydrogel content (75% HG) resulted in a greater ΔE* (83.59) compared to a 50% HG bigel (ΔE* = 69.45) when free lutein was incorporated, indicating that the proportion of hydrogel significantly modulates the visual color difference [18].

**Figure 3:**
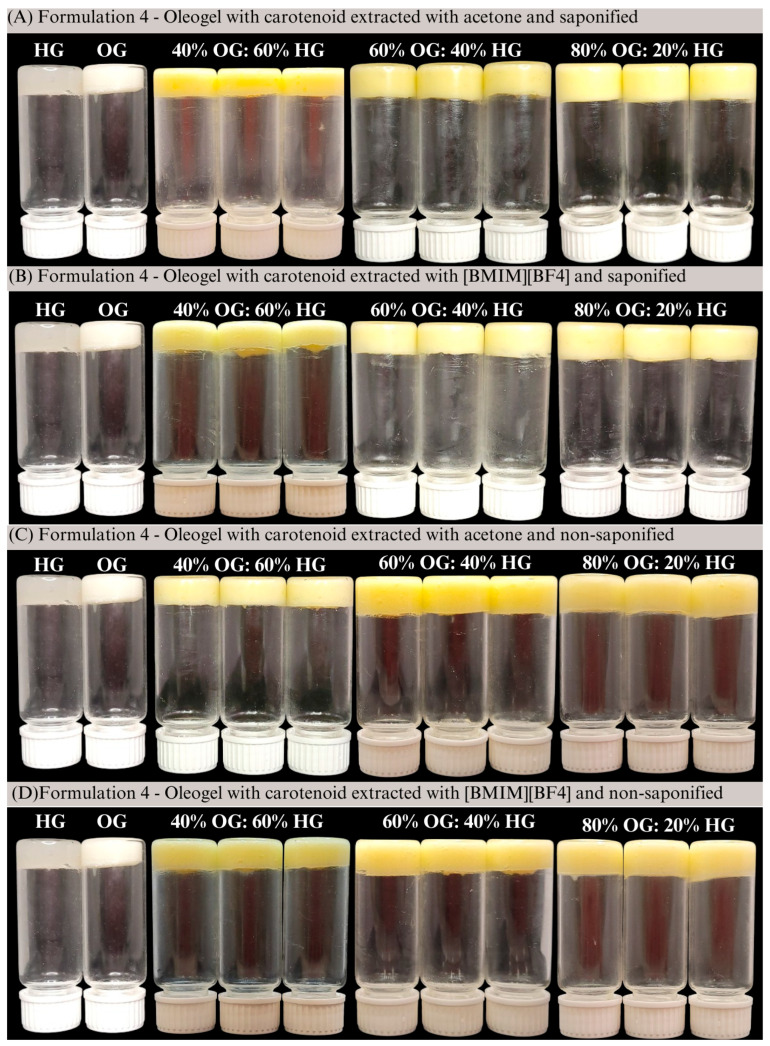
Visual appearance of carotenoid-enriched bigels (**A**) extracted with acetone and saponified; (**B**) extracted with [BMIM][BF4] and saponified; (**C**) extracted with acetone and non-saponified and (**D**) extracted with [BMIM][BF4] and non-saponified.

Overall, the evaluation of ΔE* values provided additional insights into the perceptibility of color changes in carotenoid-enriched bigels under different conditions. The results demonstrated that, while some variations were noticeable to the naked eye, particularly when comparing different extraction solvents and oleogel-to-hydrogel ratio, none of the samples exhibited ΔE* values above 10, indicating the absence of drastic color alterations, as shown in Figure 3. Differences related to crosslinking time were generally subtle, with ΔE* values remaining below 2 (range 0.25 to 1.90), reflecting minimal visual impact. On the other hand, the choice of extraction solvent resulted in a more pronounced effect, with the 80% OG bigel extracted with [BMIM][BF4] reaching a ΔE* value greater than 4. Overall, the extraction method and the oleogel-to-hydrogel ratio influenced color to some extent, although the variations remained within a range generally considered acceptable for visual perception.

## 3. Conclusions

Based on our results, formulation variables exerted differential impacts on the CIELAB color parameters of carotenoid-enriched bigels. Extraction solvent and oleogel-to-hydrogel ratio were the most influential factors, with [BMIM][BF4] extraction producing significant reductions in L*, a*, b*, and Chroma* alongside an increase in hue*, and higher OG ratio proportionally elevating L* and hue* while decreasing a*. Saponification and crosslinking time had only minor, non-significant effects. Multivariate regression confirmed these trends and additionally revealed that all factors except crosslinking time significantly modulate hue*. The notably higher ΔE* values for bigels with 80% OG extracted with [BMIM][BF4] and between the proportions of 40% vs. 60% OG suggest a more noticeable color difference. Collectively, these findings underscore the critical importance of solvent and matrix composition in tuning bigel coloration and provide a framework for the rational design of colored bioactive delivery systems.

## 4. Materials and Methods

### 4.1. Materials

Pequi fruits (*Caryocar brasiliense* Camb.) were collected in Goiânia, Brazil, and the carotenoid extraction was performed according to the method described by Ramos-Souza et al. (2023) [12]. Bleached beeswax was purchased from Bianquímica Co., Ltd. (São Paulo, Brazil), while xanthan gum (mesh 190–220) and agar (mesh 80) were obtained from Dinâmica Química Contemporânea Ltda. (São Paulo, Brazil). Commercial sunflower oil was acquired from a local supermarket. Also, the ionic liquid 1-butyl-3-methylimidazolium tetrafluoroborate ([BMIM][BF4]) was purchased from Sigma Aldrich, St. Louis, MO, USA. 

### 4.2. Pequi Carotenoid Extraction

Pequi carotenoids were extracted using acetone or the ionic liquid [BMIM][BF4], employing a solid–liquid ratio of 1:3 and a co-solvent (ethanol) ratio of 1:1 over three extraction cycles with ultrasound-assisted homogenization (550 W, 80% amplitude, 300 s). Extracts were partitioned with ethyl ether: petroleum ether (2:1). For free carotenoids, saponification was performed with 20% methanolic potassium hydroxide (KOH) for 12 h, followed by washing to remove the alkali. All extracts were concentrated to dryness under vacuum (<35 °C) and nitrogen flow for quantification [12].

### 4.3. HPLC-PDA-MS Analysis

Carotenoid extracts were analyzed using a Shimadzu High-Performance Liquid Chromatography (HPLC) system equipped with a quaternary pump (LC-20AD), degasser (DGU20A5), photodiode array detector (PAD; SPD-M20A) (Kyoto, Japan), and a mass spectrometer with an iron trap analyzer and atmospheric pressure chemical ionization (APCI) source (AmaZon Speed ETD, Bruker Daltonics, Bremen, Germany). For non-saponified carotenoids, the chromatographic conditions followed [34]. Briefly, separations were performed on a C30 YMC column (5 µm, 250 × 4.6 mm i.d., Waters, Milford, MA, USA) maintained at 35 °C. The mobile phases were solvent A (methanol: MTBE: water, 81:15:4, *v*/*v*/*v*) and solvent B (16:80.4:3.6, *v*/*v*/*v*). The linear gradient at 1.0 mL/min was as follows: 99% A to 44% A over 39 min, then to 0% A in 6 min, returned to 99% A in 5 min, and held for 5 min (total runtime: 55 min). The protocol [35] employed for saponified carotenoids using the same C30 column. The mobile phase consisted of a linear gradient of methanol and MTBE: from 95:5 to 70:30 over 30 min, and to 50:50 over 20 min, maintained for an additional 50 min. The flow rate was 0.9 mL/min, and the column temperature was 22 °C. In both analyses, UV–Visible spectra were recorded from 250 to 600 nm, with chromatograms monitored at 450 nm. APCI-MS was performed in positive ion mode with the following settings: corona current at 4000 nA, source temperature 450 °C, dry gas (N_2_) temperature 350 °C at 5 L/min, and nebulizer pressure at 60 psi. Full scan MS spectra were collected in the *m*/*z* range of 100–1200, and MS/MS data were acquired in automatic mode. Saponified and non-saponified pequi extracts were characterized by their chromatographic elution order, UV–Visible spectral characteristics (including λ_max_, spectral fine structure [%III/II], and *cis*-peak intensity [%AB/AII]), and mass spectrometric data (protonated molecular ions [M+H]^+^ and MS/MS fragmentation patterns). Carotenoids were individually quantified using analytical five-point calibration curves prepared with authentic standards of all-*trans*-lutein (1.0–50.0 μg/mL), all-*trans*-zeaxanthin (1.0–50.0 μg/mL), all-*trans*-β-cryptoxanthin (1.0–60.0 μg/mL), and all-*trans*-β-carotene (1.0–50.0 μg/mL). All calibration curves showed good linearity (r^2^ = 0.99), and the detection limit was 0.1 μg/mL. The carotenoid yields were expressed as μg/g of dry matter. Additionally, the percentage values (%) presented in the manuscript refer to the relative proportion of each carotenoid group in the total carotenoid profile, calculated from the chromatographic peak areas. These data were included to illustrate the distribution of free, and esterified xanthophylls within each extract. All data were interpreted in comparison with previously reported values in the literature [34,35,36,37].

### 4.4. Hydrogels (HG), Oleogels (OG), and Bigel Formulations

Bigel formulations were prepared following the protocol described by [16]. Briefly, the hydrogel was obtained by dissolving agar and xanthan gum in distilled water at 80 °C under stirring (2000 rpm, 10 min). The oleogel was prepared by heating bleached beeswax and soy lecithin in sunflower oil containing pequi carotenoids at 85 °C under stirring (1000 rpm, 15 min). Hydrogels and oleogels were then combined at 50 °C in three different ratios (40:60, 60:40, and 80:20 OG: HG) using a high-torque mechanical shaker (2000 rpm, 10 min) followed by homogenization with an Ultra-Turrax (IKA, Staufen, Germany) (14,000 rpm, 3 min).

### 4.5. Color on Bigels

Bigel color was evaluated using a portable spectrophotometer (CM-26d, Konica Minolta^®^, Tokyo, Japan) based on the CIELAB system, recording L* (lightness), a* (red-green), and b* (yellow-blue) values. The total color difference (ΔE*), chroma (C*), and hue angle (h*) were calculated according to Equations (1)–(3), as described by [38].

(1) $$\Delta E*=\sqrt{{\left(\Delta L*\right)}^{2}+({\Delta a*)}^{2}+{\left(\Delta b*\right)}^{2}}\;$$

(2) $$C*=\sqrt{{\left(a*\right)}^{2}+{\left(b*\right)}^{2}}$$

(3) $$h=arctan\frac{b*}{a*}$$

Color differences between the bigels were interpreted based on the classification proposed by Li et al. (2017) [33]: ΔE* ≤ 1 was considered imperceptible to the human eye; 1 < ΔE* ≤ 2 detectable only upon close observation; 2 < ΔE* ≤ 10 noticeable at a glance; 10 < ΔE* ≤ 50 representing a clear and distinct color difference; and ΔE* ≥ 50 indicating opposite colors. All experiments were performed in triplicate.

### 4.6. Bright-Field Microscopy Analysis

Bigel bright-field micrographs were characterized at room temperature (23 ± 2 °C) using a microscope (Carl Zeiss GmbH, Oberkochen, Germany) equipped with an AxioCam ICc5 digital camera supported by the AxioCam software 5S (Carl Zeiss). Bigel was carefully placed on a microscope glass slide, gently covered with a glass coverslip, and observed at a magnification of 20× and 40×. Microscopic images were analyzed using ImageJ software version 1.x.

### 4.7. Statistical Analysis

Descriptive statistics were expressed as mean ± standard deviation. Linear regression models were used to assess the effect of each independent variable, crosslinking time (1 h vs. 12 h), solvent type (acetone vs. [BMIM][BF4]), saponification (saponified vs. non-saponified), and oleogel-to-hydrogel (OG: HG) ratios (40%, 60%, and 80% OG), on each color parameter (L*, a*, b*, Chroma*, and hue*). Multiple linear regression models were also created to evaluate all factors combined and independent effects on the color parameters. Statistical significance was set at *p* < 0.05. The data was analyzed using Stata software, version 14.2, reporting regression coefficients (β), with a 95% confidence interval and a 5% significance level.

## Figures and Tables

**Table 1:** Chromatographic and UV/Vis characteristics of non-saponified pequi carotenoid extracts, obtained from HPLC-PAD-MS/MS.

			UV-Vis Characteristics		
Peak ^a^	Carotenoids	*t*_R_ (min) ^b^	λ_(max)_^c^	%III/II ^d^	%A_B_/II ^e^
1	*cis*-antheraxanthin	5.8	419, 444, 471	52	n.d.
2	all-*trans*-antheraxanthin	7.8	420, 445, 473	60	0
3	all-*trans*-zeaxanthin	10.9	423, 450, 480	25	0
4	*cis*-neoxanthin-ester	13.0	330, 417, 441, 470	82	12
5	all-*trans*-antheraxanthin-ester	13.7	421, 445, 474	60	0
6	*cis*-neoxanthin-ester	16.1	330, 417, 441, 470	81	12
7	all-*trans*-antheraxanthin-ester	19.6	421, 445, 474	60	0
8	*cis*-neoxanthin-ester	20.2	330, 417, 441, 470	81	12
9	all-*trans*-antheraxanthin-ester	23.2	421, 445, 474	60	0
10	all-*trans*-antheraxanthin-ester	27.8	421, 445, 474	60	0
11	all-*trans*-β-carotene	30.3	425, 452, 478	25	0
12	all-*trans*-antheraxanthin -ester	37.6	421, 445, 474	60	0
13	all-*trans*-antheraxanthin-ester	40.1	420, 445, 473	60	0
14	all-*trans*-antheraxanthin-ester	42.7	420, 445, 473	60	0
15	all-*trans*-zeaxanthin-ester	43.3	423, 450, 480	25	0
16	all-*trans*-zeaxanthin-ester	44.6	423, 450, 480	25	0
17	*cis*-zeaxanthin-ester	45.3	332, 421, 447, 474	20	18
18	all-*trans*-zeaxanthin-ester	45.5	423, 450, 480	25	0
19	all-*trans*-zeaxanthin-ester	46.1	423, 450, 480	25	0
20	all-*trans*-zeaxanthin-ester	46.6	423, 450, 480	25	0

^a^ Numbered according to Figure 1. ^b^ t_R_: Retention time on the C_30_ column. ^c^ Linear gradient MEOH, MTBE and water. ^d^ Spectral fine structure: Ratio of the height of the longest wavelength absorption peak (III) and that of the middle absorption peak (II). ^e^ Ratio of the *cis* peak (A_B_) and the middle absorption peak (II). n.d.: not detected.

**Table 2:** Chromatographic and UV/Vis characteristics of saponified pequi carotenoid extracts, obtained from HPLC-PAD-MS/MS.

			UV-Vis Characteristics			Mass Spectrometry Characteristics (*m*/*z*)	
Peak ^a^	Carotenoids	*t*_R_ ^b^	λ_(max)_^c^	%III/II ^d^	%A_B_/II ^e^	[M+H]	MS/MS
1	all-*trans*-neoxanthin	6.0	416, 439, 469	82	0	601	583[M+H-18]^+^, 565[M+ H-18-18]^+^, 547[M+H-18-18-18]^+^, 491[M+H-18-18-18-56]^+^, 221
2	all-*trans*-antheraxanthin	8.7	420, 444, 471	50	0	585	567[M+H-18]^+^, 549[M+H-18-18]^+^, 493[M+H-18-18-56]^+^, 221
3	9-*cis*-antheraxanthin	12.7	332, 420, 442, 470	32	18	585	567[M+H-18]^+^, 549[M+H-18-18]^+^, 493[M+H-18-18-56]^+^, 221
4	13-*cis*-zeaxanthin	15.2	336, 418, 443, 468	n.c.	43	569	551[M+H-18]^+^, 533[M+H-18-18]^+^, 463[M+H-106]^+^
5	not identified	17.6	335, 419, 441, 469	50	18	n.d.	n.d.
6	all-*trans*-zeaxanthin	18.3	423, 449, 476	25	0	569	551[M+H-18]^+^, 533[M+H-18-18]^+^, 463[M+H-106]^+^
7	*cis*-5,6-epoxy-β-carotene	19.5	332, 415, 440, 467	60	12	553	535[M+H-18]^+^, 461[M+H-92]^+^, 205
8	all-*trans*-β-cryptoxanthin	28.0	423, 450, 476	25	0	553	535[M+H-18]^+^, 495, 461[M+H-92]^+^
9	5,8-epoxy-β-carotene	32.0	402, 424, 447	40	0	553	535[M+H-18]^+^, 461[M+H-92]^+^, 205
10	all-*trans*-β-carotene	39.2	425, 451, 477	33	0	537	444[M-92]^+^

^a^ Numbered according to Figure 1. ^b^ t_R_: Retention time on the C_30_ column in minutes. ^c^ Linear gradient MEOH: MTBE. ^d^ Spectral fine structure: Ratio of the height of the longest wavelength absorption peak (III) and that of the middle absorption peak (II). ^e^ Ratio of the *cis* peak (A_B_) and the middle absorption peak (II). n.c.: not calculed.

**Table 3:** Color parameters of bigels with incorporated saponified or non-saponified pequi carotenoids extracted using acetone or [BMIM][BF4] after 1 h or 12 h of cross-linking.

Color Parameters	OG	A NS	A S	B NS	B S
L*	40% 1 h	76.15 ± 0.05	75.89 ± 0.04	73.47 ± 0.10	73.16 ± 0.08
	40% 12 h	75.15 ± 0.31	74.52 ± 0.80	72.40 ± 0.39	71.77 ± 0.64
	60% 1 h	76.91 ± 0.15	76.80 ± 0.01	74.94 ± 0.28	76.26 ± 0.04
	60% 12 h	76.96 ± 0.01	75.96 ± 0.11	75.20 ± 0.01	76.17 ± 0.01
	80% 1 h	79.04 ± 0.05	78.51 ± 0.04	77.73 ± 0.01	73.45 ± 0.01
	80% 12 h	78.52 ± 0.03	77.42 ± 0.01	76.69 ± 0.24	73.36 ± 0.01
a*	40% 1 h	0.94 ± 0.01	1.76 ± 0.01	0.28 ± 0.01	−0.10 ± 0.05
	40% 12 h	0.92 ± 0.02	1.55 ± 0.27	0.03 ± 0.16	−0.06 ± 0.03
	60% 1 h	0.44 ± 0.01	0.24 ± 0.00	−0.04 ± 0.04	−0.23 ± 0.00
	60% 12 h	0.60 ± 0.00	0.37 ± 0.01	0.10 ± 0.00	−0.31 ± 0.01
	80% 1 h	0.89 ± 0.01	−0.44 ± 0.00	−0.16 ± 0.01	0.02 ± 0.00
	80% 12 h	0.91 ± 0.02	−0.40 ± 0.01	−0.15 ± 0.05	0.00 ± 0.00
b*	40% 1 h	27.42 ± 0.03	32.26 ± 0.13	28.38 ± 0.05	27.63 ± 0.20
	40% 12 h	27.32 ± 0.09	30.95 ± 1.40	27.63 ± 0.17	28.32 ± 0.15
	60% 1 h	27.95 ± 0.02	27.71 ± 0.01	26.52 ± 0.36	24.62 ± 0.01
	60% 12 h	27.76 ± 0.00	28.12 ± 0.01	26.92 ± 0.00	24.38 ± 0.01
	80% 1 h	30.84 ± 0.03	29.48 ± 0.01	25.76 ± 0.01	28.53 ± 0.01
	80% 12 h	30.47 ± 0.06	29.21 ± 0.00	25.17 ± 0.40	27.96 ± 0.01
Chroma*	40% 1 h	27.44 ± 0.03	32.31 ± 0.13	28.38 ± 0.05	27.63 ± 0.20
	40% 12 h	27.33 ± 0.08	30.99 ± 1.41	27.63 ± 0.17	28.32 ± 0.15
	60% 1 h	27.95 ± 0.02	27.71 ± 0.01	26.52 ± 0.36	24.62 ± 0.01
	60% 12 h	27.77 ± 0.00	28.13 ± 0.01	26.92 ± 0.00	24.38 ± 0.01
	80% 1 h	30.85 ± 0.03	29.48 ± 0.01	25.76 ± 0.01	28.53 ± 0.01
	80% 12 h	30.48 ± 0.06	29.21 ± 0.00	25.17 ± 0.39	27.96 ± 0.01
hue* (°)	40% 1 h	88.04 ± 0.01	86.87 ± 0.01	89.43 ± 0.02	90.20 ± 0.10
	40% 12 h	88.07 ± 0.04	87.15 ± 0.37	89.74 ± 0.13	90.11 ± 0.07
	60% 1 h	89.10 ± 0.02	89.50 ± 0.00	90.09 ± 0.08	90.38 ± 0.00
	60% 12 h	88.76 ± 0.00	89.24 ± 0.01	89.79 ± 0.00	90.38 ± 0.02
	80% 1 h	88.35 ± 0.01	90.86 ± 0.00	90.35 ± 0.01	89.96 ± 0.00
	80% 12 h	88.29 ± 0.03	90.78 ± 0.01	90.34 ± 0.11	90.00 ± 0.00

Values are expressed as the median ± standard deviation (n = 3). OG—Oleogel; Ac NS—carotenoids extracted with acetone and non-saponified; Ac S—carotenoids extracted with acetone and saponified; B NS—carotenoids extracted with [BMIM][BF4] and non-saponified; B S—carotenoids extracted with [BMIM][BF4] and saponified.

**Table 4:** Linear regression model evaluating the effects of crosslinking time, extraction solvent, and saponification on the CIELAB color parameters of carotenoid-enriched bigels.

Color Parameter	Crosslinking (1 vs. 12 h)			Solvent (Acetone vs. [BMIM][BF4])			Saponification Process		
	*β*	*CI*	*p*	*Β*	*CI*	*p*	*β*	*CI*	*p*
L*	−0.681	−1.60; 0.238	0.144	−2.269	−3.030; −1.509	0.000	0.830	−0.082; 1.743	0.074
a*	−0.003	−0.281; 0.275	0.986	−0.699	−0.922; −0.476	0.000	0.140	−0.136; 0.417	0.315
b*	−0.240	−1.156; 0.675	0.602	−2.304	−3.039; −1.569	0.000	−0.586	−1.493; 0.321	0.202
Chroma*	−0.237	−1.157; 0.682	0.608	−2.321	−3.057; −1.584	0.000	−0.594	−1.505; 0.316	0.197
hue*	−0.065	−0.588; 0.458	0.805	1.339	0.925; 1.753	0.000	−0.282	−0.801; 0.237	0.282

**Table 5:** Linear regression model evaluating the effects of oleogel-to-hydrogel ratios on the CIELAB color parameters of carotenoid-enriched bigels.

Color Parameter	Effect of Different Oleogel-to-Hydrogel Ratios ^1^		
	*Β*	CI	*p*
L*			
60% OG	2.087	1.168; 3.006	0.000
80% OG	2.774	1.85; 3.692	0.000
a*			
60% OG	−0.520	−0.827; −0.213	0.001
80% OG	−0.582	−0.889; −0275	0.000
b*			
60% OG	−1.990	−2.998; −0.981	0.000
80% OG	−0.311	−1.319; 0.698	0.541
Chroma*			
60% OG	−2.009	−3.021; −0.997	0.000
80% OG	−0.329	−1.341; 0.683	0.518
hue*			
60% OG	0.991	0.421; 1.561	0.001
80% OG	1.164	0.594; 1.734	0.000

^1^ The effects were evaluated using 40% OG formulation as the reference level.

**Table 6:** Multivariate regression model evaluating the combined effects of crosslinking time, extraction solvent, saponification, and oleogel-to-hydrogel ratios on the CIELAB color parameters of carotenoid-enriched bigels.

Color Parameter	L*			a*			b*			Chroma*			hue*		
Variable	*β*	*CI*	*p*	*β*	*CI*	*p*	*β*	*CI*	*p*	*β*	*CI*	*p*	*β*	*CI*	*p*
Crosslinking after 12 h	−0.659	−1.121; −0.197	0.006	0.003	−0.183; 0.188	0.978	−0.254	−0.863 0.356	0.409	−0.251	−0.860; 0.359	0.414	−0.075	−0.408; 0.258	0.654
Solvent [BMIM][BF4]	−2.291	−2.753; −1.829	0.000	−0.704	−0.889; −0.519	0.000	−2.290	−2.900; −1.681	0.000	−2.307	−2.916; −1.697	0.000	1.349	1.016; 1.682	0.000
Non-saponified carotenoids	0.787	0.324; 1.250	0.001	0.181	−0.005; 0.366	0.056	−0.488	−1.098; 0.123	0.116	−0.495	−1.105; 0.116	0.110	−0.362	−0.695; −0.0288	0.034
60% OG *	2.054	1.488; 2.620	0.000	−0.527	−0.754; −0.300	0.000	−1.969	−2.716; −1.222	0.000	−1.989	−2.735; −1.242	0.000	1.006	0.598; 1.413	0.000
80% OG *	2.741	2.175; 3.307	0.000	−0.589	−0.816; −0.362	0.000	−0.291	−1.038; 0.457	0.440	−0.309	−1.055; 0.438	0.412	1.179	0.771; 1.586	0.000

* The effects of oleogel: hydrogel ratios were evaluated using 40% OG formulation as the reference level.

## Data Availability

The original contributions presented in this study are included in the article. Further inquiries can be directed to the corresponding author.

## Abbreviations

The following abbreviations are used in this manuscript:

Ac NS	Carotenoids extracted with acetone and non-saponified
Ac S	Carotenoids extracted with acetone and saponified
B NS	Carotenoids extracted with [BMIM][BF4] and non-saponified
B S	Carotenoids extracted with [BMIM][BF4] and saponified
[BMIM][BF4]	1-butyl-3-methylimidazolium tetrafluoroborate
CI	Confidence Interval
HG	Hydrogel
HPLC	High-Performance Liquid Chromatography
KOH	Potassium hydroxide
MS	Mass spectrometry
NS	Non-saponified
OG	Oleogel
S	Saponified
PDA	photodiode array detector
UV/Vis	Ultraviolet–Visible spectral characteristics
